# Does Pharmacological Treatment Reduce the Incidence of Lower Urinary Tract Symptoms (LUTS) after Transobturator Sling?

**DOI:** 10.1155/2019/7271289

**Published:** 2019-03-06

**Authors:** Tomasz Rechberger, Andrzej Wrobel, Alicja Zietek, Ewa Rechberger, Beata Kulik-Rechberger, Michal Bogusiewicz, Pawel Miotla

**Affiliations:** ^1^2^nd^ Department of Gynecology, Medical University of Lublin, Lublin, Poland; ^2^Department of Paediatric Propedeutics, Medical University of Lublin, Lublin, Poland

## Abstract

**Aim:**

Lower urinary tract symptoms (LUTS) frequently affect patients immediately after midurethral sling (MUS) placement. The objective of the study was to assess if solifenacin or mirabegron decreases incidence of LUTS in women who underwent transobturator MUS.

**Methods:**

A prospective randomized trial was conducted on patients undergoing ambulatory transobturator midurethral sling due to stress urinary incontinence (SUI). All participants were questioned before and after surgery for occurrence of bothersome LUTS. A total of 328 patients who underwent transobturator MUS were randomly assigned to one of three groups: prophylaxis with 10 mg of solifenacin, prophylaxis with 50 mg of mirabegron, or without any additional treatment. LUTS evolution and efficacy of solifenacin and mirabegron were analyzed based on results of assessments made during follow-up visits at 1 and 6 weeks after surgery. Comparison of the prevalence of LUTS was done using chi^2^ test.

**Results:**

Prevalence of urgency and frequency episodes increased notably 1 week after sling placement and then came down to baseline levels. Solifenacin and mirabegron significantly reduced the incidence of urgency after 1 week, but after 6 weeks the beneficial effect was observed only in case of solifenacin. Treatment with mirabegron reduced the percentage of patients suffering from frequency after 6 weeks. Although prevalence of nocturia did not raise after sling placement, both treatments significantly reduced the incidence of this complaint after 6 weeks. Pharmacological treatment did not modulate the course of hesitancy and terminal dribbling.

**Conclusions:**

Treatment with solifenacin or mirabegron may significantly reduce the incidence of undesired LUTS after MUS.

## 1. Introduction

Lower urinary tracts symptoms (LUTS) encompass a broad group of symptoms affecting proper storage of urine and effective self-controlled urination. These bothersome symptoms are categorized as storage, voiding, or postvoiding [1]. The steadily increased number of midurethral sling procedures performed to treat female stress urinary incontinence with or without concomitant prolapse surgery has resulted in a definite rise in the number of iatrogenic LUTS caused by anatomical obstructions of the urethra [2]. Moreover, even without obvious bladder outlet obstruction, a majority of women after sling procedures are transiently suffering from undesired LUTS [3]. In fact, reported rates of various voiding dysfunction vary between the different sling placement techniques, which include retropubic and transobturator sling passage and the different sling materials used, as well as patient and surgeon factors [4]. Even if, after sling surgery, the long-term retention rate (defined as catheter dependency for >28 days after surgery) varies between 1 to 10%, other voiding or storage dysfunctions are generally underreported—probably due to the fact that happy-to-be-dry patients might not be compelled to report minor voiding dysfunctions [5].

Keeping this in mind, there is no doubt that a considerable percentage of the LUTS in women encountered after sling procedures are, in fact, iatrogenic in nature [6]. The temporal relationship between surgery and the onset of new LUTS is the most important diagnostic issue [4]. These new undesired symptoms can vary tremendously and can be as nonspecific as urgency and frequency, spraying and splitting, or findings of low-flow voiding on noninvasive uroflowmetry with increase of PVR in sonographic investigation. Even if surgeons and patients should anticipate a period of transient voiding dysfunction during postoperative recovery, every effort should be undertaken in order to decrease the percentage of undesired LUTS after incontinence surgery and, subsequently, to increase patient satisfaction.

Since the LUTS after MUS procedures are very bothersome for the patients and negatively affect their quality of life, we tested the hypothesis whether short-term prophylaxis with solifenacin or mirabegron introduced in the very early postoperative period may alter the incidence of postoperative LUTS.

## 2. Materials and Methods

The study protocol was approved by our local institutional ethical committee and all patients gave written informed consent before inclusion. Out of 630 patients with stress urinary incontinence treated in our department from October 2014 to January 2018, 345 agreed to participate in this study. Women were eligible for the study if they had symptoms of SUI as assessed via a positive cough test either in the supine or standing positions at bladder volume of approximately 250-300 ml and had a voiding frequency of 7 times or less per day, a bladder capacity ≥250 ml, postvoid residual (PVR) ≤ 50 ml without clinically relevant pelvic organ prolapse (POP-Q ≤1) [7]. Study exclusion criteria were the evidence of obstructed voiding in the absence of prolapse and previous pelvic surgery. Patients were questioned before and after surgery for occurrence of storage symptoms (urgency, increased day time frequency, nocturia) as previously described [3]. In patients who reported urgency at baseline, urodynamic testing was performed to exclude detrusor overactivity, and only those without detrusor overactivity during filling cystometry were included in this study. We consider the presence of undesired urgency if occurred at least 3 times daily or more before micturition but without uncontrolled urinary leakage. Nocturia was defined as 2 or more voiding episodes during nighttime.

Based on these criteria, the study was conducted on a group of 328 women who underwent an ambulatory transobturator midurethral sling (MUS) procedure with additional tape fixation as previously described [8]. Patients received a day preceding surgery single dose (3 gram) of fosfomycin trometamol orally as a standardized antibiotic protocol.

**Figure 1:**
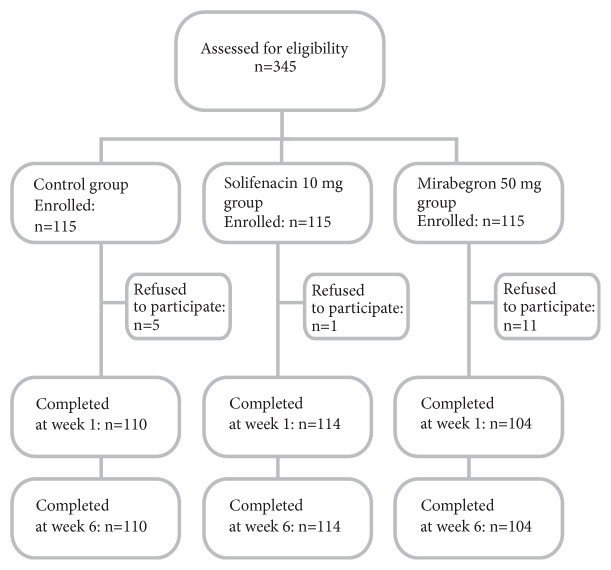
Flowchart of the participants in the study.

Before discharge, the patients were assessed via ultrasonography (postvoid residual and tape position) and uroflowmetry to exclude the possibility of bladder outlet obstruction. Simple randomization was used from pseudorandom numbers generated by a computer to allocate patients into the study groups in a ratio of 1:1:1. Investigators A.Z. and E.R. were not involved in the surgical procedures, but they were responsible for the randomization process. After randomization, but before surgery, 17 patients resigned from participation in this study: 5 from control group, 1 from the solifenacin group, and 11 from the mirabegron group (Figure 1). The remaining patients were then allocated into 3 study groups:

1. without any additional treatment (control group, n=110),
2. prophylaxis with 10 mg of solifenacin taken orally once daily for 4 weeks (n=114),
3. prophylaxis with 50 mg of mirabegron taken orally once daily for 4 weeks (n=104).

Follow-up visits were conducted by phone-call at one week, while an office-based examination occurred at 6 weeks after surgical intervention.

The sample size calculation was based on a previous study showing 52% incidence of urgency 1 week after sling placement [3]. To detect the decrease of urgency incidence by half, the sample size required for an alpha 0.05 and a power of 90% was 70 participants.

Statistical analyses were performed with Statistica package version 12.0 (StatSoft Inc., Tulsa, OK, USA). A p value <0.05 was considered statistically significant. The Chi-squared test was used as statistical test applied to sets of categorical data to evaluate how likely it is that any observed difference between the sets arose by chance. Interim analysis of data obtained from 65 patients in the control group and 56 in the treatment group showed that, for urgency occurrence, 50 participants in each group would be enough to reach more than the 95% power of chi^2^ at a 2-sided significance level of 0.05 for each group. For comparison of continuous variables (age, BMI, parity) ANOVA with post hoc tests and the Student's t test were applied. Continuous variables are presented as the mean ± SD.

## 3. Results

Demographic and clinical data did not differ between investigated groups (Table 1).

**Figure 2:**
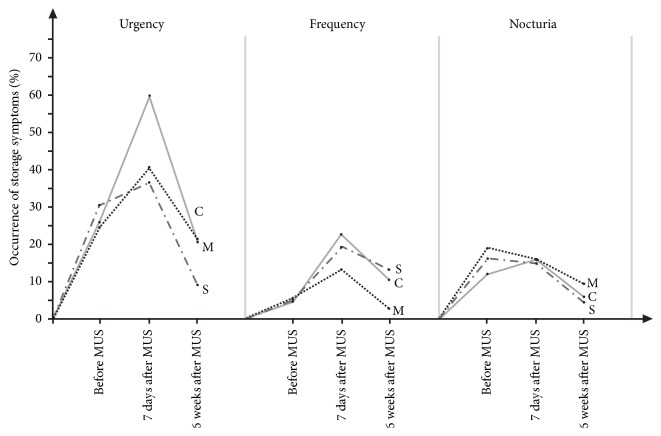
The evolution of storage symptoms after midurethral sling surgery in control (C), treatment with mirabegron 50 mg (M), and treatment with solifenacin 10 mg (S).

At baseline, the incidence of LUTS did not differ significantly between the investigated groups. The evolution of storage symptoms in all studied groups is presented in Figure 2.

In all groups, the occurrence of urgency rose significantly 1 week after sling placement and then came down to baseline levels, or, as in the solifenacin group at the end of the study, was lower, in comparison with baseline evaluation. Both treatment regimens significantly reduced the incidence of urgency after 1 week, but after 6 weeks, this beneficial effect was observed only in case of solifenacin (Table 2).

Similarly to urgency, in all groups, the incidence of frequency rose noticeably after 1 week. At week 6, the percentage of patients suffering from this complaint was significantly higher in the control group and lower in the mirabegron group when compared to baseline (Table 3).

We did not observe any increase in the incidence of nocturia after sling placement. In fact, in all groups a notable drop in the prevalence of this symptom was found at final assessment. At week 6 in both treatment groups, the incidence of nocturia was significantly lower in comparison with the baseline. Indeed, comparisons between study groups did not show any significant differences (Table 4). In contrast, incidence of voiding symptoms (hesitancy, terminal dribbling) rose noticeably after MUS and remained more frequent at week 6, when compared to baseline evaluation. Pharmacological treatment, either with solifenacin or mirabegron, did not modulate the course of these symptoms (Table 5).

## 4. Discussion

Over the last decade, a dramatic rise in the use of midurethral synthetic slings has been reported due to its high clinical efficacy accompanied by technical simplicity, and minimal patient morbidity. However, the increase in midurethral procedures that has been observed around the world is accompanied by a varied proportion of de novo postoperative voiding dysfunctions manifested by increased voiding times, decreased maximum flow rates (Qmax), increased mean detrusor pressure (Pdet), increased detrusor pressure at maximal flow, increased mean urethral resistance, and elevated postvoid residual volumes [9–12].

Moreover, many women, even with urodynamic stress incontinence, often demonstrate other lower urinary tract symptoms (LUTS) including frequency, nocturia, and urgency [13]. The estimated prevalence of these symptoms among women suffering from urinary incontinence varies from 29 to 69% [14]. Nevertheless, most studies on SUI solely focus on the cure of incontinence after midurethral sling placement rather than the effect of these procedures on coexistent or de novo arising LUTS. To the best of our knowledge, this is the first randomized trial focused on active pharmacological prevention of undesired LUTS after transobturator sling placement among SUI sufferers.

The ideal timing of an intervention for undesired voiding dysfunctions after incontinence surgery has not been clearly established since transient voiding dysfunction, including urinary retention, may be seen during postoperative recovery. In our preliminary recently published study, we clearly show that in the first 6 weeks after MUS, more than 60% of all women will experience some undesired LUTS which negatively influence their quality of life [3]. As these LUTS are probably inherently connected with this type of surgical intervention, all patients should be informed that such undesired symptoms could occur in the first few weeks after intervention, but will probably undergo natural resolution within few months after surgery [15]. In the past, several attempts have been undertaken in order to predict the probability of voiding dysfunction occurrence after sling placement based on preoperative and intraoperative variables. Even if, as in the SISTEr trial, sole focus was upon patients with dysfunctional emptying after sling procedure, the authors of such studies did not identify any preoperative predictors of voiding dysfunction, whereas other studies have suggested that altered preoperative detrusor contractility can predict postoperative sling obstruction with concomitant unwanted LUTS [16–19].

In the TOMUS trial, iatrogenic obstruction leading to LUTS following sling surgery was more likely to occur after retropubic slings placement rather than the transobturator route, although, overall, this was an uncommon event [19]. High-grade pelvic organ prolapse might also have a role in post-op voiding dysfunction by contributing to voiding obstruction, but this was not the case in our study since we only included patients without concomitant POP [20].

The prevalence of iatrogenic LUTS after MUS surgery is very common regardless of type of surgery, yet, symptoms severity, including urinary retention, urgency, urgency incontinence, hesitancy, straining to void, weak stream, nocturia, frequency, and UTI, could be different depending on the study population. The method of treatment can vary according to physician or patient preference and can include temporary intermittent catheterization, indwelling catheterization, pharmacological management, biofeedback therapy, urethral dilation, and office-based sling loosening [4].

Based on the literature data, it seems that in a majority of patients, some transient undesired LUTS after MUS procedures are simply unavoidable; however, simple, short-term pharmacological intervention can decrease the percentage nearly by half. There is no doubt that the best solution would be to identify patients with increased risks of developing undesired LUTS after MUS before the operation and, only in these, introduce prophylaxis with either anticholinergics or mirabegron. Still, as clearly shown before, the patient's preoperative history is only minimally useful in the identification of women at increased risk for the development of urgency, with the exception of the complaint of increased daytime frequency, which is a very common symptom among SUI patients [21].

It was also shown that the finding of increased detrusor pressure during the filling phase of cystometry on preoperative conventional urodynamics in particular may help identify (but, not at 100%) women at increased risk for postoperative de novo urge incontinence following a minimally invasive midurethral sling procedure. Nevertheless, proper identification of patients at increased risk of developing undesired LUTS after surgery would help to individualize preoperative counseling regarding expected outcomes and patient's satisfaction with their surgical procedure [22].

We understand that our study also has several limitations. First of all the single setting of this randomized trial and the exclusion of patients who had underwent any other types of midurethral slings (retropubic or single incision). The obvious limitation is also the fact that patients were aware of types of pharmacological intervention since we used pharmaceuticals already available on the market and that we tested only one antimuscarinic medication. The reasons for our choice of solifenacin were that this drug is reimbursed in Poland and it has relatively good clinical efficacy in reducing urgency, with relatively low side effects when compared to other antimuscarinics [23]. On the other hand, the strengths of our study were the prospective nature of this trial and the relatively large number of women in the study (exceeding by almost twice the calculated number in order to reach statistical power of the study). Of note, all participants had undergone standardized preoperative evaluation using standard ICS recommendations, met standard high-quality operative procedures performed by a high volume experienced surgeon (T.R.), and experienced continuous follow-up almost without drop-out.

## 5. Conclusions

Based on the results of this study, we can conclude that short-term pharmacological treatment (4 weeks) either with anticholinergic (solifenacin) or *β*-3-adrenergic receptor agonist (mirabegron) could significantly decrease the percentage of undesired LUTS (namely, urgency and frequency) after transobturator midurethral sling surgery.

## Figures and Tables

**Table 1:** Demographic characteristics of patients groups.

Variable	Control group (n=110)	Treatment group 1 (10 mg of solifenacin) (n=114)	Treatment group 2 (50 mg of mirabegron) (n=104)
Age (years)	55.5 (±11.3)	54.6 (±13.1)	53.6 (±12.2)

BMI (kg/m^2^)	27.3 (±3.3)	27.0 (±3.7)	26.8 (±4.2)

Postmenopausal n, (%)	73 (66.4)	69 (60.5)	61 (58.7)

Parity	1.9 (±1.0)	1.9 (±1.0)	1.7 (±1.2)

There was no statistically significant difference between all investigated groups.

**Table 2:** The evolution of urgency in the course of the study.

Variable	Baseline n (%) [B]	Week 1 n (%) [W1]	Week 6 n (%) [W6]	Statistical analyses inside each group
Control group (n=110) [C]	29 (26.4)	66 (60.0)	23 (20.9)	B *vs. *W1, *p* <0.001 B vs. W6, NS W1 *vs. *W6, *p* <0.001

Treatment group 1 (10 mg of solifenacin) (n=114) [S]	35 (30.7)	43 (37.7)	11 (9.7)	B *vs. *W1, NS B vs. W6, *p *< 0.001 W1 *vs. *W6, *p* < 0.001

Treatment group 2 (50 mg of mirabegron) (n=104) [M]	26 (25)	43 (41.3)	23 (22.1)	B *vs. *W1, *p*<0.05 B vs. W6, NS W1 *vs. *W6, *p*<0.005

Baseline: C vs. S (NS); C vs. M (NS); S vs. M (NS).

Week 1: C vs. S (*p* <0.001); C vs. M (*p *<0.001); S vs. M (NS).

Week 6: C vs. S (*p*<0.05); C vs. M (NS); S vs. M (*p*<0.05).

**Table 3:** The evolution of frequency in the course of the study.

Variable	Baseline n (%) [B]	Week 1 n (%) [W1]	Week 6 n (%) [W6]	Statistical analyses inside each group
Control group (n=110) [C]	5 (4.5)	25 (22.7)	12 (10.9)	B *vs.*W1, *p* <0.001 B vs. W6, NS W1 *vs. *W6, *p* <0.05

Treatment group 1 (10 mg of solifenacin) (n=114) [S]	6 (5.3)	22 (19.3)	12 (10.5)	B *vs.*W1, *p* <0.005 B vs. W6, NS W1 *vs. *W6, NS

Treatment group 2 (50 mg of mirabegron) (n=104) [M]	6 (5.8)	15 (13.6)	3 (2.9)	B *vs.*W1, *p* <0.05 B vs. W6, NS W1 *vs. *W6, *p* <0.005

Baseline: C vs. S (NS); C vs. M (NS); S vs. M (NS).

Week 1: C vs. S (NS); C vs. M (NS); S vs. M (NS).

Week 6: C vs. S (NS); C vs. M (*p*<0.05); S vs. M (*p* <0.05).

**Table 4:** The evolution of nocturia in the course of the study.

Variable	Baseline n (%) [B]	Week 1 n (%) [W1]	Week 6 n (%) [W6]	Statistical analyses inside each group
Control group (n=110) [C]	16 (14.5)	18 (16.4)	8 (7.3)	B *vs.*W1, NS B vs. W6, NS W1 *vs. *W6, *p* <0.05

Treatment group 1 (10 mg of solifenacin) (n=114) [S]	19 (16.7)	17 (14.9)	5 (4.4)	B *vs.*W1, NS B vs. W6, *p* <0.005 W1 *vs. *W6, *p* <0.01

Treatment group 2 (50 mg of mirabegron) (n=104) [M]	20 (19.2)	17 (16.3)	10 (9.6)	B *vs.*W1, NS B vs. W6, *p* <0.05 W1 *vs. *W6, NS

Baseline: C vs. S (NS); C vs. M (NS); S vs. M (NS).

Week 1: C vs. S (NS); C vs. M (NS); S vs. M (NS).

Week 6: C vs. S (NS); C vs. M (NS); S vs. M (NS).

**Table 5:** The evolution of hesitancy and terminal dribbling.

Hesitancy					Terminal dribbling			
Variable	Baseline n (%) [B]	Week 1 n (%) [W1]	Week 6 n (%) [W6]	Statistical analyses inside each group	Baseline n (%) [B]	Week 1 n (%) [W1]	Week 6 n (%) [W6]	Statistical analyses inside each group
Control group (n=110) [C]	11 (10)	45 (40.9)	29 (26.4)	B vs W1 chi^2^=27.7 p<0.001 B vs W6 chi^2^= 9.9 p=0.017 W1 vs W6 chi^2^ = 5.2 p =0.022	2 (1.8)	43 (39.1)	23 (20.9)	B vs W1 chi^2^=47 p<0.001 B vs W6 chi^2^= 19.9 p<0.0001 W1 vs W6 chi^2^ = 8.7, p =0.0033

Treatment group 1 (10 mg of solifenacin) (n=114) [S]	11 (10.6)	48 (46.2)	32 (30.8)	B vs W1 chi^2^=15.8 p<0.001 B vs W6 chi^2^=5.2 p=0.022 W1 vs W6 NS	2 (1.9)	48 (46.2)	32 (30.8)	B vs W1 chi^2^=31.1 p<0.001 B vs W1 chi^2^=23.5 p<0.0001 P1 vs P2 NS

Treatment group 2 (50 mg of mirabegron) (n=104) [M]	13 (11.4)	38 (33.3)	26 (22.8)	B vs W1 chi^2^ =32.4 p<0.001 B vs W6 chi^2^= 12.9 p=<0.0001 W1 vs W6 chi^2^=5.2 p=0.023	2 (1.8)	32 (28.1)	26 (22.8)	B vs W1 chi^2^ =57.1 p<0.001 B vs W6 chi^2^= 31.6 p<0.0001 W1 vs W6 chi^2^=5.2 p=0.023

Baseline: C vs. S (NS); C vs. M (NS); S vs. M (NS).

Week 1: C vs. S (NS); C vs. M (NS); S vs. M (NS).

Week 6: C vs. S (NS); C vs. M (NS); S vs. M (NS).

Pharmacological treatment did not modulate the course of these symptoms.

## Data Availability

The data used to support the findings of this study are included within the article.
